# Sensitivity and specificity of faecal tumour M2 pyruvate kinase for detection of colorectal adenomas in a large screening study

**DOI:** 10.1038/sj.bjc.6604427

**Published:** 2008-06-10

**Authors:** U Haug, S Hundt, H Brenner

**Affiliations:** 1 Division of Clinical Epidemiology and Aging Research, German Cancer Research Center, Bergheimer Str. 20, Heidelberg 69115, Germany

**Keywords:** faecal tumour M2 pyruvate kinase, colorectal adenomas, screening, stool testing

## Abstract

The measurement of faecal tumour M2 pyruvate kinase (tumour M2 PK) has been proposed as a novel approach for early detection of colorectal cancer (CRC). However, as regards the potential of the test to detect precursors to CRC, an issue that is highly relevant to estimate its use in reducing CRC incidence and mortality, the available evidence is scant and controversial. The aim of our study was to determine the performance characteristics of the tumour M2 PK test with respect to colorectal adenomas in the target population of screening. Among 1082 participants of screening colonoscopy in Germany, of whom 30% had any adenoma and 10% had an advanced adenoma, the median (interquartile range) tumour M2 PK level in the whole study population was 1.3 U ml^−1^ (0.3–3.3). At a cutoff value of 4 U ml^−1^, sensitivity was 22 and 23% for detection of advanced and other adenomas, respectively, whereas specificity was 82%. The area under the receiver-operating characteristics curve (95% confidence interval) was 0.54 (0.51–0.58) and 0.56 (0.52–0.59) for advanced and other adenomas, respectively. In conclusion, the tumour M2 PK test has only very limited potential to distinguish between people bearing precursors to CRC and people with no finding at colonoscopy.

The measurement of faecal tumour M2 pyruvate kinase (tumour M2 PK), an isoform of the glycolytic enzyme PK, which is overexpressed in proliferating cells such as tumour cells, has been proposed as a novel approach for early detection of colorectal cancer (CRC) (Mazurek *et al*, 2002). Previous studies investigating this test reported a comparably high sensitivity for CRC ranging about 70–80%, whereas specificity ranged about 70–90% (Hardt *et al*, 2004; Vogel *et al*, 2005; Shastri *et al*, 2006; Tonus *et al*, 2006; Haug *et al*, 2007; Mulder *et al*, 2007; Koss *et al*, 2008). However, the potential of the tumour M2 PK test to detect colorectal adenomas, an issue that is highly relevant for estimating its use in reducing CRC incidence and mortality, has been investigated rarely and with rather limited sample size only (Vogel *et al*, 2005; Shastri *et al*, 2006; Mulder *et al*, 2007; Koss *et al*, 2008). Interpretation of pertinent findings regarding relevance for screening is further limited in that previous studies were typically conducted in subjects referred to colonoscopy because of symptoms or special risk factors. The aim of our study was to determine performance characteristics of the tumour M2 PK test with respect to colorectal adenomas in a large sample of women and men from the target population of screening.

## Materials and methods

The analyses were part of the BliTz study (Begleitende Evaluierung innovativer Testverfahren zur Darmkrebsfrüherkennung), an ongoing screening study conducted in cooperation with 20 gastroenterological practises in South-West Germany since January 2006, which aims at the comparative evaluation of new tests for early detection of CRC. The study includes participants of screening colonoscopy, which is offered by the German health-care system since October 2002 to average-risk subjects aged 55 years or older. People with visible colorectal bleeding or with insufficient knowledge of the German language were excluded. After written informed consent, patients scheduled for screening colonoscopy were asked to provide a stool sample before bowel preparation for colonoscopy. The stool sample was collected according to the manufacturer's instructions with a special stick capturing 4 mg of stool and was sent by mail to a central laboratory. The sampling date and the date of receipt at the laboratory were documented. Upon receipt, stool samples were stored at −20°C and analysed for tumour M2 PK within 1 month. After colonoscopy was performed, reports on colonoscopic and histological findings were collected and information was extracted in a standardised manner. The study was approved by the Ethics Committee of the University of Heidelberg.

Tumour M2 PK was measured with a commercially available sandwich ELISA (ScheBo® Biotech AG, Giessen, Germany) based on monoclonal antibodies against dimeric M2 PK. All analyses were done in a blinded manner and under standardised conditions.

For the present analyses, study participants tested for tumour M2 PK until 10 December 2007 with adequate bowel preparation and complete colonoscopy (caecum was reached) were included. We excluded patients with CRC (this subgroup comprised only 10 participants by the end of 2007) as sensitivity with respect to CRC will be analysed separately after continued recruitment of a much larger number of screening participants. To account for the limited stability of tumour M2 PK at room temperature (Haug *et al*, 2006), only participants whose stool samples arrived at the laboratory within 2 days after sampling were included as recommended by the manufacturer. Using SAS version 9.1, sensitivity, specificity and predictive values of the test for detecting any adenoma and, more specifically, advanced adenomas (i.e., adenomas that are at least 1 cm in diameter, adenomas with villous components or with high-grade dysplasia) and other adenomas were calculated at a cutoff value of 4 U ml^−1^, the cutoff level proposed by the manufacturer, and 95% confidence intervals (95% CI) were determined based on the exact binomial distribution. Nonparametric and *χ*^2^ tests were used to compare medians and proportions, respectively. In addition, sensitivity and specificity were derived at a broad range of alternative cutoff values and, receiver-operating characteristic curves (ROC) were constructed to visualise the discriminatory power of the test. Area under the curves (AUC) and corresponding 95% CIs were calculated using MedCalc for Windows, version 9.3.9.0.

## Results

Overall, 1082 participants (mean age 63 years, 50% females) were included in the analyses. Although the majority of stool samples arrived at the laboratory within 1 day, 32% of samples arrived after 2 days only. The median (interquartile range) tumour M2 PK level in the whole study population was 1.3 U ml^−1^ (0.3–3.3). Median tumour M2 PK levels did not differ by gender or duration of mailing, but tended to be higher in older age groups (*P*=0.002) (see Table 1).

The distribution of findings at colonoscopy, median tumour M2 PK and test performance characteristics at a cutoff level of 4 U ml^−1^ (recommended by the manufacturer) by subgroup are shown in Table 2. Overall, about 30% of participants had at least one adenoma and about 10% of participants had an advanced adenoma. Sensitivity (95% CI) for advanced adenomas and other adenomas was 22% (14–31%) and 23% (17–29%), respectively. There was no significant variation of sensitivity by location, number or size of adenomas. Sensitivity appeared to be slightly higher among participants with three or more adenomas, but CIs were wide given the small number of patients in this group. Sensitivity did not vary by age, gender or shipping time of stool samples. The positive predictive value (95% CI) was 34% (28–41%) for bearing any adenoma and 11% (7–16%) for bearing an advanced adenoma.

Specificity (95% CI) was about 82% (78–84%), irrespective of whether participants with hyperplastic polyps were included as controls or not. Specificity did not vary in gender or shipping time of stool samples, but tended to be lower in older age groups (*P*=0.001). The negative predictive value was 61% (57–64%) when including only participants with no polyp as controls and 71% (68–74%) when including participants with hyperplastic or no polyp as controls.

**Figure 1:**
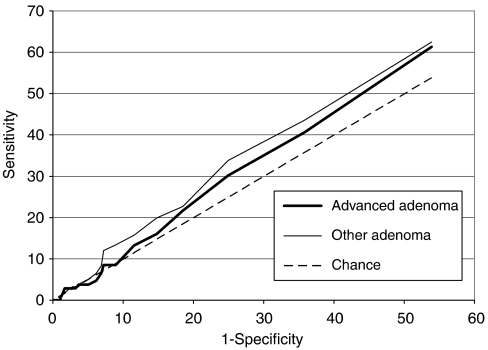
Receiver-operating characteristics curve for the tumour M2 PK stool test in detecting patients with colorectal adenomas.

The AUC (95% CI) was 0.54 (0.51–0.58) for advanced adenomas and 0.56 (0.52–0.59) for other adenomas (see Figure 1).

## Discussion

Our study, which is the largest study so far to investigate the potential of the tumour M2 PK test to detect colorectal adenomas, showed a sensitivity for both advanced and other adenomas of about 22–23%, which was only slightly higher than the false-positive rate in the same study population. Thus, despite the AUCs being slightly but statistically significantly above 0.50, this test appears to have only very limited potential to distinguish between people bearing precursors to CRC and people with negative colonoscopy.

The results of previous studies investigating this issue, which relied on smaller sample sizes (number of adenomas <50 in all studies) and study populations typically recruited in clinical settings, were ambiguous: although two of them were in line with our findings (Vogel *et al*, 2005; Shastri *et al*, 2006), one study reported a statistically significant difference in positivity rates between subjects with adenomas and with no findings (Mulder *et al*, 2007), whereas another study also reported a difference, but were unable to allow for meaningful statistical comparison (only 10 subjects with adenomas and 13 controls) (Koss *et al*, 2008).

The finding that faecal tumour M2 PK levels are not markedly increased in subjects with colorectal adenomas is in contrast to the much higher overall sensitivity that has been reported for invasive CRC, but it is consistent with the stage-dependent performance of this test observed for CRC: all pertinent studies reported notably higher sensitivity for more advanced CRC than for less advanced CRC (Hardt *et al*, 2004; Tonus *et al*, 2006; Haug *et al*, 2007; Mulder *et al*, 2007; Koss *et al*, 2008). This suggests that in the precancerous phase, the critical stage of neoplasia leading to increased faecal tumour M2 PK levels may not be reached yet.

In our study, higher tumour M2 PK levels were observed in older age groups than in younger age groups, which slightly affected the specificity of the test but not its sensitivity. So far, only one study has investigated tumour M2 PK levels according to age, which, however, did not observe such an association (Haug *et al*, 2007). Further evidence is therefore needed to confirm a potential age dependency of this marker.

Our study might be limited by the diagnostic accuracy of colonoscopy regarding the detection of precancerous lesions in routine practise (Barclay *et al*, 2006). In particular, 20 different gastroenterologists were involved in patient recruitment and colonoscopy. However, high levels of qualification and experience are a prerequisite for conducting screening colonoscopies in Germany: only experienced endoscopists (internists/gastroenterologists or surgeons with pertinent certified specialisations, having conducted at least 200 colonoscopies and at least 50 polypectomies under supervision in the preceding two calendar years) are permitted to conduct screening colonoscopies. Requirements for maintenance of permission include conduction of at least 200 colonoscopies and at least 10 polypectomies per year. Conduction of a study in this very setting should ensure maximum possible relevance of results under routine screening conditions.

In conclusion, the tumour M2 PK test has only very limited potential to distinguish between people bearing precursors to CRC and people with no finding at colonoscopy in the screening setting.

## Figures and Tables

**Table 1:** Distribution of age and sex among study participants and median tumour M2 PK levels by age

**Age group (years)**	**No. of participants (%)**	**% females**	**Median tumour M2 PK level (interquartile range) (U ml^−1^)**
30–49	23 (2.1)	52.2	0.8 (0.1–1.4)
50–59	356 (33.1)	53.1	1.0 (0.2–2.6)
60–69	517 (48.0)	51.1	1.4 (0.4–3.4)
70–79	171 (15.9)	43.3	1.8 (0.5–4.1)
80+	10 (0.9)	10.0	2.0 (1.1–8.7)

Tumour M2 PK=tumour M2 pyruvate kinase.

**Table 2:** Distribution of findings at colonoscopy, tumour M2 PK levels and performance characteristics of the tumour M2 PK test at a cutoff level of 4 U ml^−1^ among participants of the BliTz study

**Finding at colonoscopy^a^**	**No. of participants (%)**	**Median tumour M2 PK level (interquartile range) (U ml^−1^)**	**Sensitivity (95% CI) (%)**
	1082 (100.0)	1.3 (0.3–3.3)	
Advanced adenomas^b^	106 (9.8)	1.6 (0.5–3.7)	21.7 (14.3–30.8)
Other adenomas	216 (20.0)	1.7 (0.5–3.7)	22.7 (17.3–28.9)
			
*Stratified by location of adenomas* c			
Proximal	123 (38.2)^d^	1.7 (0.6–3.4)	27.8 (16.5–41.6)
Distal	145 (45.0)^d^	1.5 (0.5–3.6)	22.1 (15.6–29.7)
Both	54 (16.8)^d^	1.7 (0.3–4.3)	20.3 (13.6–28.5)
			
*Stratified by number of adenomas*			
1 adenoma	207 (64.3)^d^	1.5 (0.5–3.4)	19.8 (14.6–25.9)
2 adenomas	76 (23.6)^d^	1.8 (0.4–4.0)	23.7 (14.7–34.8)
3 or more adenomas	39 (12.1)^d^	1.8 (0.4–5.1)	33.3 (19.1–50.2)
			
*Stratified by size of adenomas (in diameter)*			
<1 cm	254 (79.0)^d^	1.5 (0.4–3.6)	22.1 (17.1–27.7)
⩾1 cm	68 (21.0)^d^	1.8 (0.6–4.0)	23.5 (14.1–35.4)
			
Hyperplastic polyp	111 (10.3)	1.0 (0.3–2.8)	NA
No polyp	649 (60.0)	1.2 (0.3–3.0)	81.5 (78.3–84.4)
No or hyperplastic polyp	760 (70.2)	1.2 (0.3–3.0)	81.7 (78.8–84.4)

BliTz=Begleitende Evaluierung innovativer Testverfahren zur Darmkrebsfrüherkennung; CI=confidence interval; NA=not applicable; tumour M2 PK=tumour M2 pyruvate kinase.

a Allocation to subgroups according to the most advanced finding at colonoscopy.

b Defined as adenomas that are at least 1 cm in diameter, adenomas with villous components or with high-grade dysplasia.

c Proximal/distal to the splenic flexure.

d Percentage in parentheses refers to participants bearing adenomas, i.e., *N*=322 participants.
